# Evaluation of Olive Pruning Effect on the Performance of the Row-Side Continuous Canopy Shaking Harvester in a High Density Olive Orchard

**DOI:** 10.3389/fpls.2019.01631

**Published:** 2020-01-15

**Authors:** António Bento Dias, José M. Falcão, Anacleto Pinheiro, José O. Peça

**Affiliations:** ^1^ Departmento de Engenharia Rural, Instituto de Ciências Agrárias e Ambientais Mediterrânicas (ICAAM), University of Évora, Évora, Portugal; ^2^ Torre das Figueiras Sociedade Agrícola Lda, Monforte, Portugal

**Keywords:** canopy, shaker, olive, pruning, performance

## Abstract

In 2009, the Side-Row Continuous Canopy Shaking Harvester project was set to develop such technology. The prototype comprises two symmetrical harvesters trailed by a farm tractor. Each harvester has a vibratory rotor with flexible rods, a catching platform with conveyors belts delivering fruits to a temporary storage bag. The removal efficiency of canopy shakers are influenced by factors like shaking frequency, ground speed as well as the dimension and shape of olive canopy. In 2014 authors started a trial to evaluate the influence of pruning in olive yield and in the performance of the Side-Row Continuous Canopy Shaking Harvester. The trial was established in an irrigated olive orchard of Picual cultivar planted in 1996 with the array 7 m x 3.5 m. In a randomised complete block design with three replications, four treatments are being compared leading to 12 plots with 30 trees/plot. The treatments under study are: T1—manual pruning using chain saws, in 2014 and 2017; T2—mechanical pruning: topping and hedging the two sides of the canopy, followed by manual pruning complement to remove wood suckers inside the canopy, in 2014 and 2017; T3—mechanical pruning: topping the canopy parallel to the ground and hedging southeast side of the canopy in 2014 and 2017; topping the canopy in July 2015 (summer pruning); hedging northwest side in winter 2016; T4—mechanical pruning: topping and hedging the two sides of the canopy in 2014 and 2017; topping the canopy in July 2015 (summer pruning). Regarding to olive yield per tree, significant differences were found among treatments on different years. However, no significant differences were found regarding the average olive yield per tree, over the period of 2014–2017. Regarding to the olive removal efficiency, only in 2016, significant differences were found among treatments on different years. No significant differences were found regarding the average of the olive removal efficiency, over the period of 2014–2017.

## Introduction

In Portugal there are currently 40,000 ha of high density olive groves (200 to 500 trees per hectare), mostly irrigated. Despite the recent diffusion of the super high density olive grove, still there will be more than 1.5 million ha of high density olive groves worldwide.

Olive harvest in high density olive orchards is usually performed by a tractor mounted trunk shaker and a canvas manually placed on the ground under the tree. Less labor demanding solutions based on inverted umbrellas linked to the trunk shaker have limited use since trees are very closely spaced to allow the umbrella to open.

Only changing from a discrete trunk shaking to a continuous canopy shaking principle will improve working capacity and will reduce the dependency over scarce and expensive labor. Grape and coffee over-the-row canopy harvesters could be used with good results in young intensive olive orchards not higher than 2.5 to 3.5 m or wider than 2 m (Ravetti and Robb, 2010). The same authors reported harvest efficiencies of 86 to 96% with a Colossus straddle harvester in an 8 years old olive grove, in Australia. This over-the-row machine is too heavy and expensive, hardly suitable to the difficult wet soil conditions encountered in the Mediterranean countries. A row-side, instead of over-the-row, concept imposing fewer limitations on tree growth is a technique bound for intensive orchards and may even be adequate for the large trees of the traditional non-irrigated orchards (Castro-García et al., 2011). Peterson (1998) designed and tested a prototype for continuous harvesting of oranges based on the side-by-side canopy shaker principle. Ferguson et al. (2002) used a side-by-side canopy prototype to harvest table olives with 90% harvest efficiency in the center of the canopy, but with significant efficiency losses in the leading and trailing edges of the canopy. The use of a canopy shaker prototype in traditional olive groves with large canopy trees showed lower efficiency than the obtained with trunk shakers (Sola-Guirado et al., 2014). In the prototype used by Sola-Guirado et al. (2016) it was found that an increase in vibration frequency or vibration amplitude enhances harvest efficiency. In 2009 the authors started a research project to develop the Side-Row Continuous Canopy Shaking Harvester—SRCCSH (Peça et al., 2014), which has been intensively tested ever since.

Cultivar, tree shape, canopy density, and pruning affect mechanical harvesting efficiency with canopy shakers (Ferguson et al., 2010).

It has been reported (Ferguson and Castro-García, 2014) that with adequate mechanical pruning, a canopy shaker harvester in table olives has provided greater harvest efficiency than manual harvesting of manually pruned trees, although this year there was a significant decrease in olive production in trees subjected to mechanical pruning. The same authors also reported that in a hedgerow olive grove, the application of mechanical pruning did not lead to significant differences in olive production compared to manual pruning. Harvesting efficiency with side-by-side canopy shaker in the mechanically pruned trees did not differ significantly from that of manual harvesting in manually pruned trees (Ferguson and Castro-García, 2014).

This paper presents and discusses the results of research conducted on a 20-year-old intensive olive grove to evaluate the influence of mechanical pruning on olive production and the performance of the SRCCSH.

## Materials and Methods

### Olive Orchard

The high density olive orchard (HD) used in the trial was established in 1996 in Herdade da Torre das Figueiras in the Alentejo region of southern Portugal (lat. 39°03’34.04’’ N; 07°28’22.00’’W). This drip irrigated HD olive orchard of Picual cultivar was installed in an array of 7 m x 3.5 m.

The orchard was planted on Chromic Luvisol soil (FAO). This region is semi-arid with strong continental influence and an annual rain mean of 500 mm concentrated in the winter.

The orchard is drip irrigated twice a week, from May till October, receiving annually an estimated volume of 1,500–2,000 m^3^/ha.

The HD olive orchard was sprayed to control olive leaf spot [*Flusicladium oleaginum (Castagne) Ritschel & U. Braun*], olive moth (*Prays oleae* Bernard), olive fly (*Bactrocera oleae* Gmelin.*)* and olive anthracnose (*Colletotrichum acutatum* Simmons *or Colletotrichum gloeosporioides* Penz.*)*. Weed control was done spraying glyphosate in the rows and with a shredder between rows. About 80 units of nitrogen, 30 units of phosphorus, and 50 units of potassium were applied to the soil and by drip irrigation in average by year.

### Equipment

**Figure 1:**
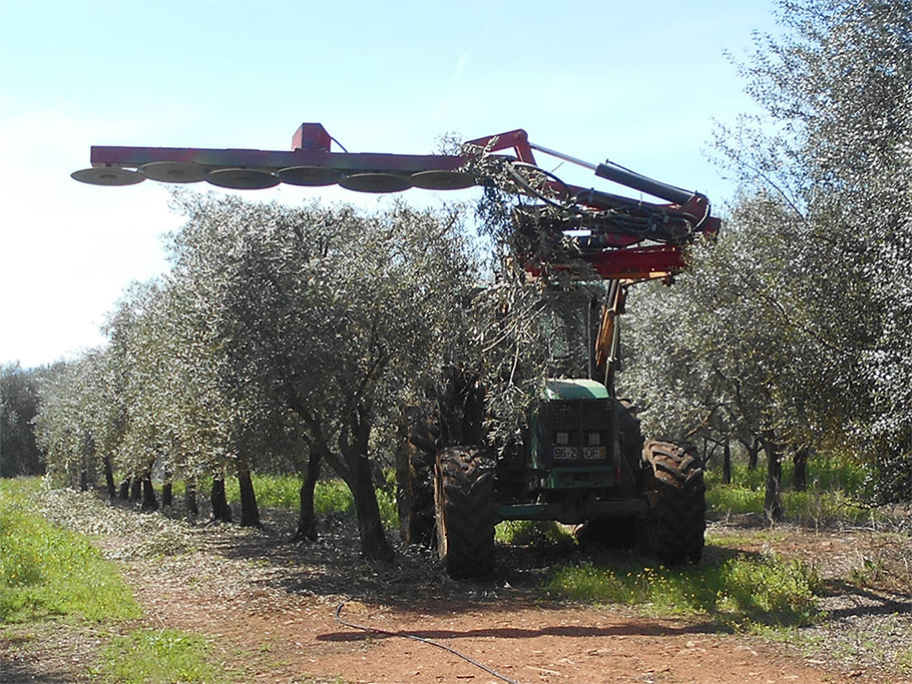
Pruning machine mounted in a tractor.

Mechanical pruning was performed using an R&O (Reynolds & Oliveira Ltd.) disk-saw pruning machine (**Figure 1**), with a 3.0 m cutting bar (Peça et al., 2002), mounted on a front loader of a 97 *kW* (DIN) 4WD agricultural tractor.

The manual pruning complement to the mechanical pruning was executed by telescopic chain saws.

**Figure 2:**
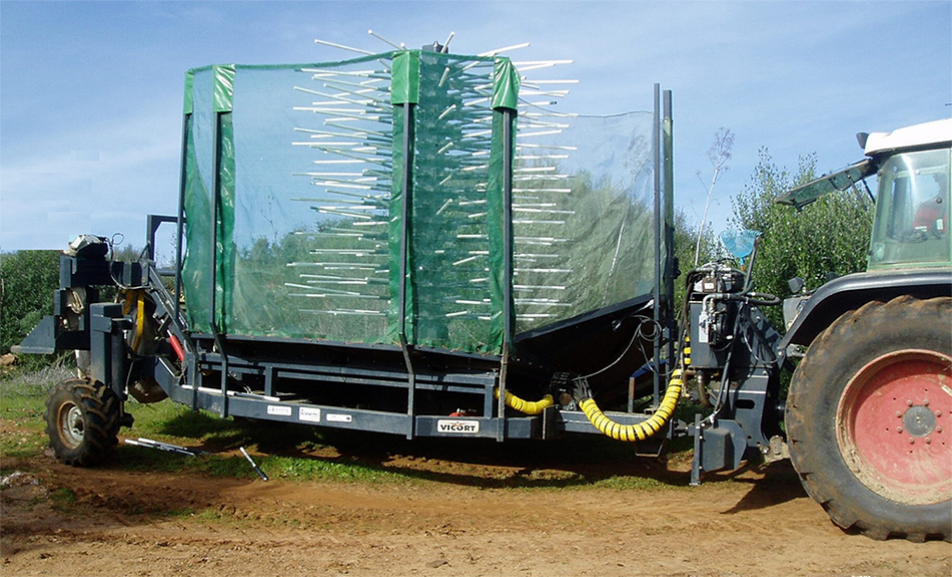
Row side continuous canopy shaker.

The SRCCSH is a prototype (**Figure 2**) developed to remove fruits from the tree brunches, collect, and transport the fruits to temporary storage (Peça et al., 2014).

The SRCCSH is based on two symmetrical machines, each one trailed by a farm tractor, moving alongside a same tree row, harvesting both sides of the trees. Fruit removal is made by a vibratory rotor with flexible rods for engaging and shaking the olive bearing branches. Vibration frequency of the vibratory rotor can be altered adjusting the tractor power-take-off speed. Removed olives are collected on a platform and conveyed to a temporary storage bag.

Operational parameters of SRCCSH were adjusted at the beginning of the harvesting season (**Table 1**).

**Table 1:** Operation parameters of Side-Row Continuous Canopy Shaking Harvester.

Harvesting season	2014	2015	2016	2017
Ground speed (*km/h*)	0.6	0.6	0.6	0.75
PTO of left unit (*rpm*)	430	430	540	610
PTO of right unit (*rpm*)	430	500	540	540

### Treatments

Four treatments (T1, T2, T3, T4), shown in **Table 2**, are being compared in a randomized complete block design with three replications leading to 12 plots, of one line each, with 30 trees per plot.

**Table 2:** Treatments: pruning interventions sequence.

Treatment	2014	2015	2016	2017
T1	Manual			Manual
T2	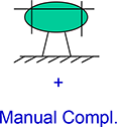			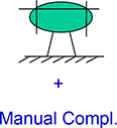
T3	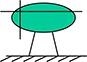	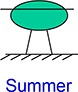	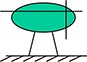	
T4	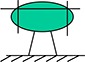	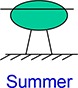		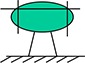

Manual, manual pruning; Manual Compl., manual pruning complement; Summer, summer pruning.

Treatment 1 (T1)—manual pruning performed in 2014 and 2017. One man per tree performed selective cuts with a chainsaw to control canopy dimension, removing branches with excessive growth, either overhanging the canopy toward the space between rows or taller than the vibratory mast of the SRCCSH.
Treatment 2 (T2)—mechanical pruning followed by a manual pruning complement was made in 2014 and 2017. Mechanical pruning consisted on a horizontal cut (topping) at the uppermost part of the canopy and a vertical cut on each side of the tree (hedging). Topping was done at approximately 3.6 m (2014) and 3.3 m (2017) in height, from the ground; hedging was done at approximately 1.8 m from the tree trunk. Manual pruning complement was performed to remove wood suckers inside the canopy and also wood stumps on each side of canopy.
Treatment 3 (T3)—mechanical pruning performed each year. In 2014, trees were topped (as in T2) followed by a vertical cut (hedging) of the southeastern side of the canopy. In July 2015 a summer topping at 3.6 m height (from the ground) was done to control growth suckers developed in uppermost part of the canopy after 2014 topping. In March 2016 the northwestern side of the canopy was hedged at a distance of 2.0 m from the tree trunk. In March 2017, trees were topped at 3.3 m height (from the ground) and hedged on the southeastern side of the canopy at a distance of 1.8 m from the tree trunk.
Treatment 4 (T4)—mechanical pruning performed in 2014, 2015, and 2017. In 2014, trees were topped (as in T2) and hedging each side of the canopy. In July 2015, a summer topping at 3.6 m height was done to control growth suckers developed at the uppermost part of the canopy, after 2014 topping. In March 2017 a horizontal cut was made at 3.3 m height followed by hedging both sides of the canopy at a distance of 1.8 m from the tree trunk.

### Assessments

Pruning operations were timed to calculate the work rates. Tree measurements of the height of the tree from the ground, width of the canopy and the distance from the base of the canopy to the ground were recorded in five trees randomly selected in each plot. Measurements were done in the same trees in the entire period of tests (2014 to 2017) and taken before and after pruning interventions or in early spring for the non-pruning years.

The mass of olives caught by the SRCCSH was measured weighing the bags from each plot.

The evaluation of the mass of olive removed but not caught by the harvester was done weighing the fruits collected on canvas placed under a group of three olive trees at three locations randomly selected in each plot.

To quantify the mass of olives not removed by the harvester, all trees in each plot were vibrated by a trunk shaker complemented by manual harvest with poles.

Total yield per tree was obtained adding the mass of olives caught by the harvester (in the bags) to the mass of olives dropped to the ground (on the canvas) plus the mass left on the tree

Harvest efficiency was calculated as follows:

$$Harvest\;efficiency\;\left(\%\right)=\frac{\text{Mass of olives caught per tree}}{\text{Total yield per tree}}$$

One-way analysis of variance were performed to annual data and general linear model univariate analysis for average data, using IBM SPSS version 24 software. Mean separation was performed by Multiple Range Duncan test at 5 and 10% significance level.

## Results

### Pruning Work Rate

**Figure 3:**
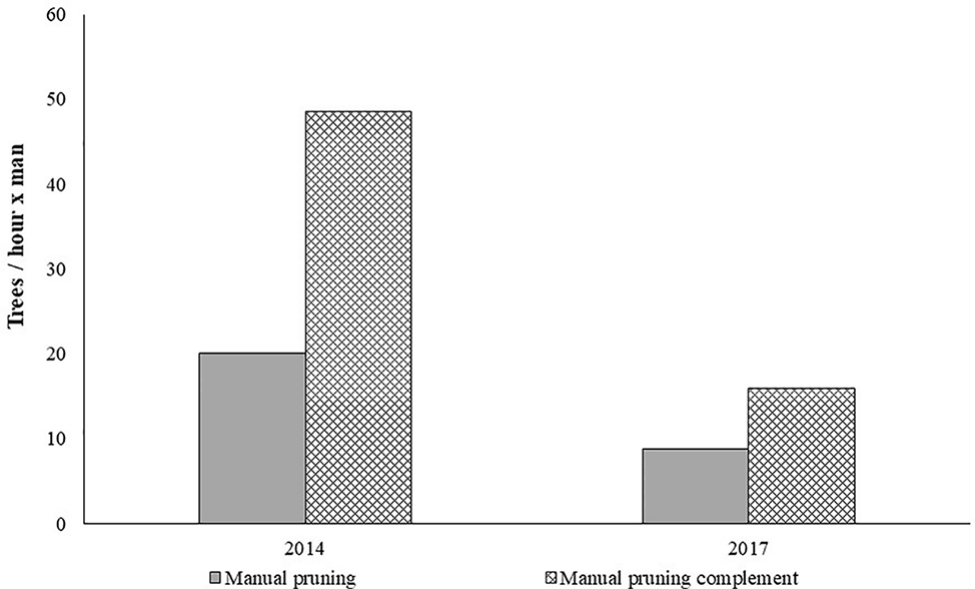
Average work rates of manual pruning.

**Figure 3** shows the manual pruning work rates obtained in 2014 and 2017. More severe pruning interventions justify a reduction in the work rate in 2017.

To avoid low work rates of manual pruning complement reported in previous research work (Peça et al, 2002) a clarification of what should consist the manual pruning complement and continuously monitoring the pruning workers led to higher work rates compared with strictly manual pruning intervention.

**Figure 4:**
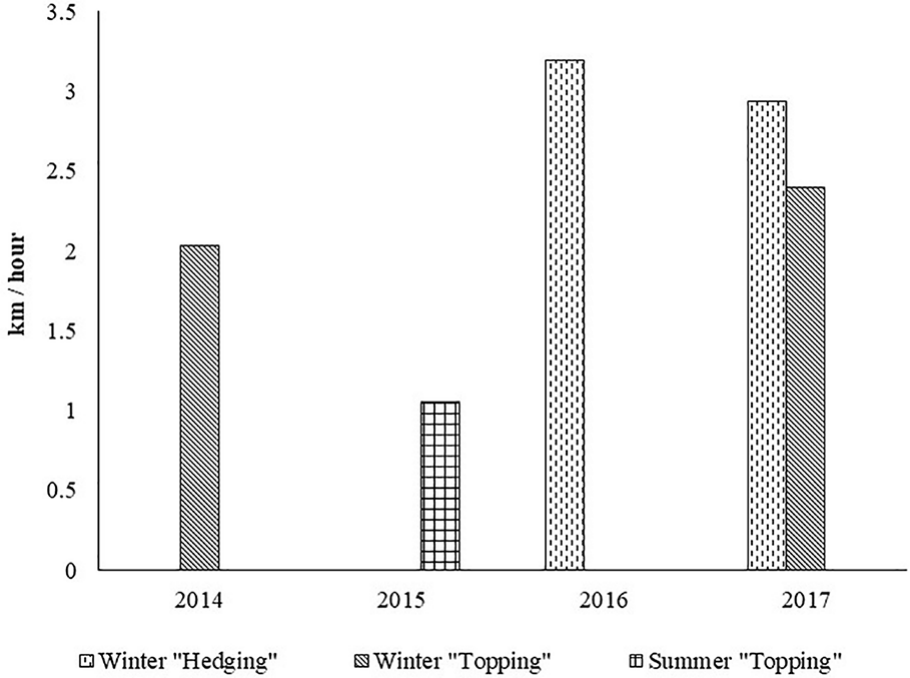
Estimated speed of advance of the tractor with the disk pruning machine is shown as a function of the type of cut.

In **Figure 4** an estimation of the speed of advance of the tractor with the disk pruning machine is shown as a function of the type of cut.

Lateral cuts of the canopy allow the tractor to move faster since the volume of leaf mass that is eliminated is smaller than in the horizontal cuts of the upper part of the canopy. In the horizontal cut the tractor has to move slower to reduce the risk of leaf and branches mass accumulating in front of the cutter bar, locking the cutter disks.

Summer cuts tend to require the tractor to move even slower, since the branches to be cut are highly flexible and tend to bend in front of the disk saw, aggravated when disks are not conveniently sharped.

**Figure 5:**
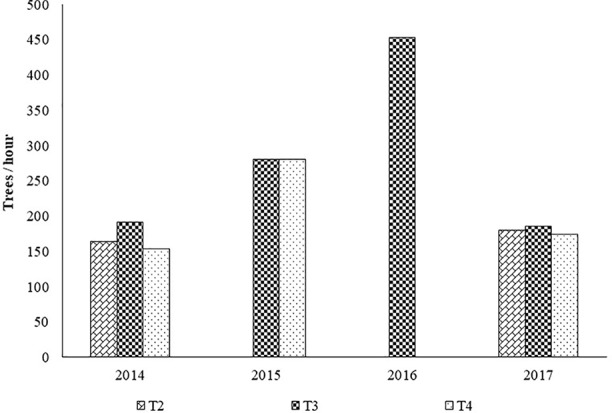
Average work rates (trees/hour x man) by treatment with the disk saw pruning machine. Legend: T1, treatment 1; T2, treatment 2; T3, treatment 3; T4, treatment 4.

**Figure 5** shows the working rate of the disk pruning machine in each treatment.

Topping and hedging on both faces corresponds to a working capacity of 155 to 180 trees per hour.

Topping and hedging of only one side did not show a higher work rate (186 to 192 trees per hour) because of the non-productive return path required to restart the work in the same direction.

Summer topping despite being carried out at a lower working speed than the winter topping, registered a greater working capacity, since only one machine pass was necessary for each row of trees, whereas two passes were required in winter topping due to wider canopies and limited cutter bar width.

### Pruning Costs

Based on the working capacities presented in **Figures 3** and **5**, the pruning costs were determined. The following assumptions were considered: 70 €/h for contractor work charges for mechanical pruning; 70 €/man-day for contractor work charges for manual pruning; 7.5 h of effective work per day.

**Figure 6:**
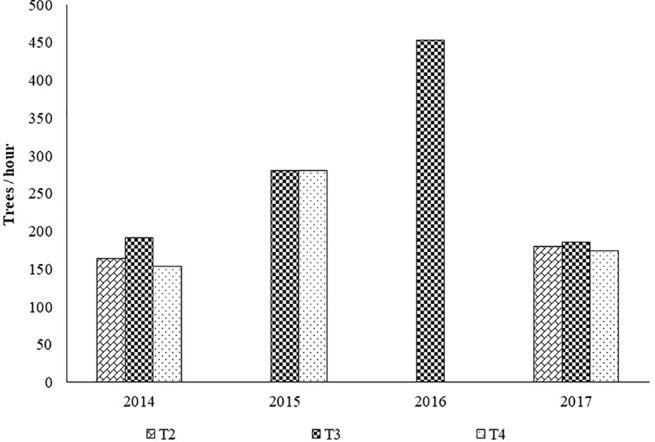
Pruning costs per treatment. Legend: T1, treatment 1; T2, treatment 2; T3, treatment 3; T4, treatment 4.

**Figure 6** shows the cost of pruning in each year and by treatment, showing also the total value per treatment over the period 2014 to 2017.

In 2014, the interventions with the disk pruning machine, comprising topping and hedging of both sides (T4) had a cost similar to that of strictly manual pruning (T1).

When, in addition to topping and hedging, a manual complement (T2) is added, pruning became more expensive than strictly manual pruning (T1).

In 2017, as a result of a more severe pruning intervention, the work rate was lower compared to what had occurred in 2014. As a consequence, the cost weight of manual pruning gave rise to higher pruning costs in T1 and T2 treatments than in T3 and T4 treatments.

Concerning total values, the cost of strictly mechanical pruning was lower than pruning with manual interventions.

### Canopy Dimensions

#### Tree Height

**Figure 7:**
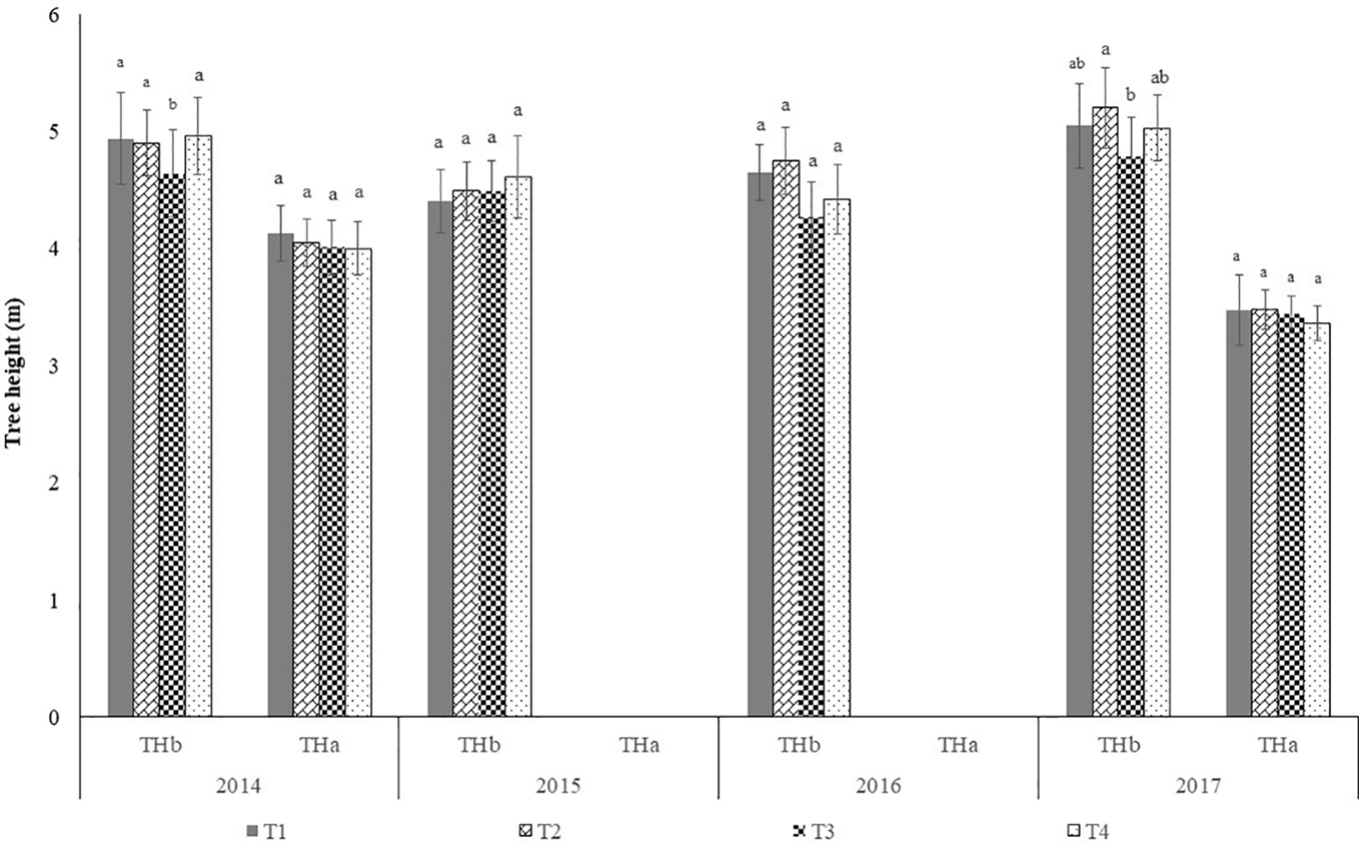
Tree height before and after winter pruning in each year (mean±sd). Legend: THb, tree height before winter pruning; THa, tree height after winter pruning; T1, treatment 1; T2, treatment 2; T3, treatment 3; T4, treatment 4. In each year, before and after pruning, columns followed by the same letter are not significantly different by Duncan multiple range test at the 5% level.

**Figure 7** shows the height of the trees, per year and treatment, before and after winter pruning. It shows the extent of the reduction in height as well as the recovery observed from one pruning intervention to the next. In treatment 3 and treatment 4 the recovery in tree height over the entire period of 2014 up to 2017 are influenced by the pruning interventions made in the summer of 2015.

The height of the trees is a relevant dimension in that the highest rods of the vibratory mast of the SRCCSH are at approximately 3.6 m above the ground.

#### Canopy Width

**Figure 8:**
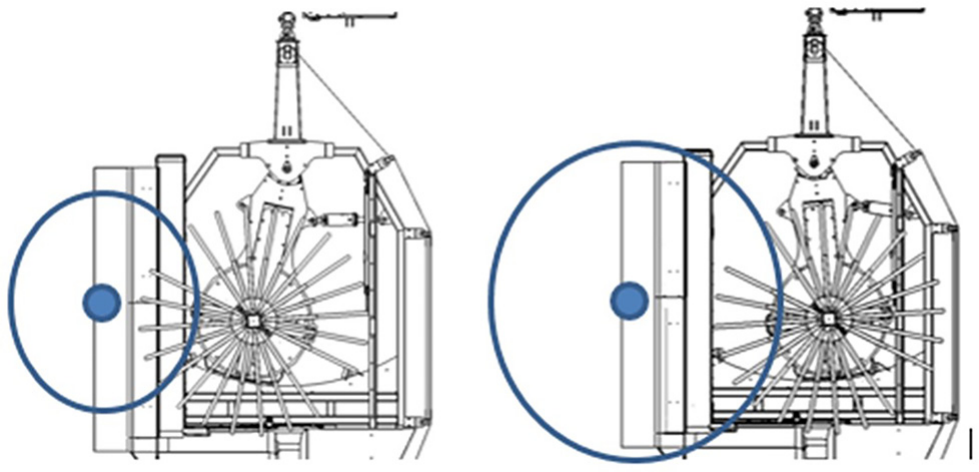
Top view of the Side-Row Continuous Canopy Shaking Harvester showing lateral position of the vibratory mast.

This dimension is relevant in that, once the interface of the SRCCSH has been positioned in relation to the trunk of the olive tree, the rods of the vibratory mast must penetrate the canopy, leaving the steel guiding tubes of the rods outside the canopy. Since the SRCCSH allows lateral placement of the vibratory mast, **Figure 8**, adequate canopy width limits should be between 1.5 and 3.6 m.

**Figure 9:**
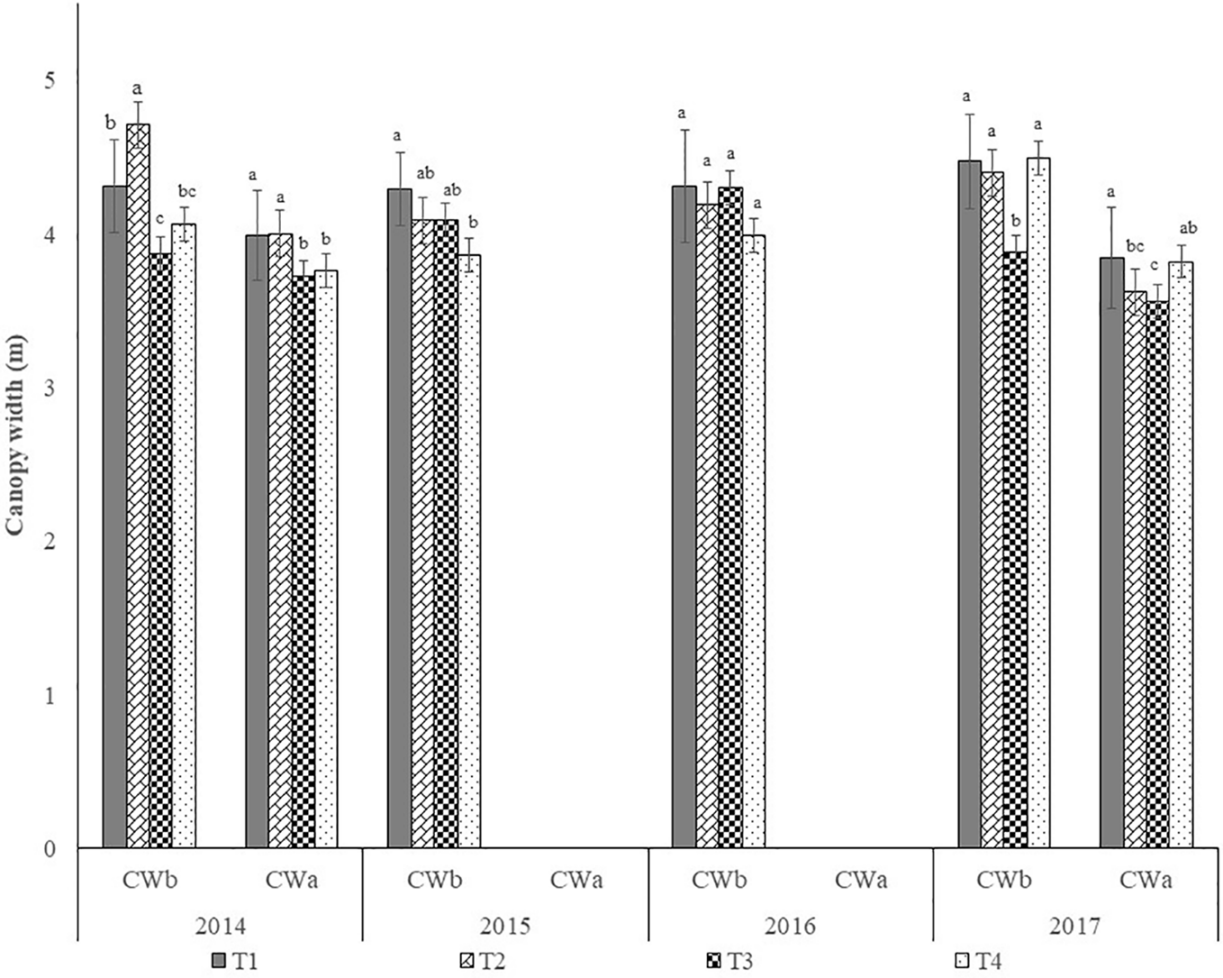
Canopy width before and after pruning in each year (mean±sd). Legend: CWb, canopy width before winter pruning; CWa, canopy width after winter pruning; T1, treatment 1; T2, treatment 2; T3, treatment 3; T4, treatment 4. In each year, before and after pruning, columns followed by the same letter are not significantly different by Duncan multiple range test at the 5% level, with exception of CWa in 2014 and 2017, where p≤0.1.

**Figure 9** shows the average canopy width of the trees, per year and treatment, before and after winter pruning. It shows the extent of the reduction in canopy width as well as the recovery observed from one pruning intervention to the next.

### Olive Yield

**Figure 10:**
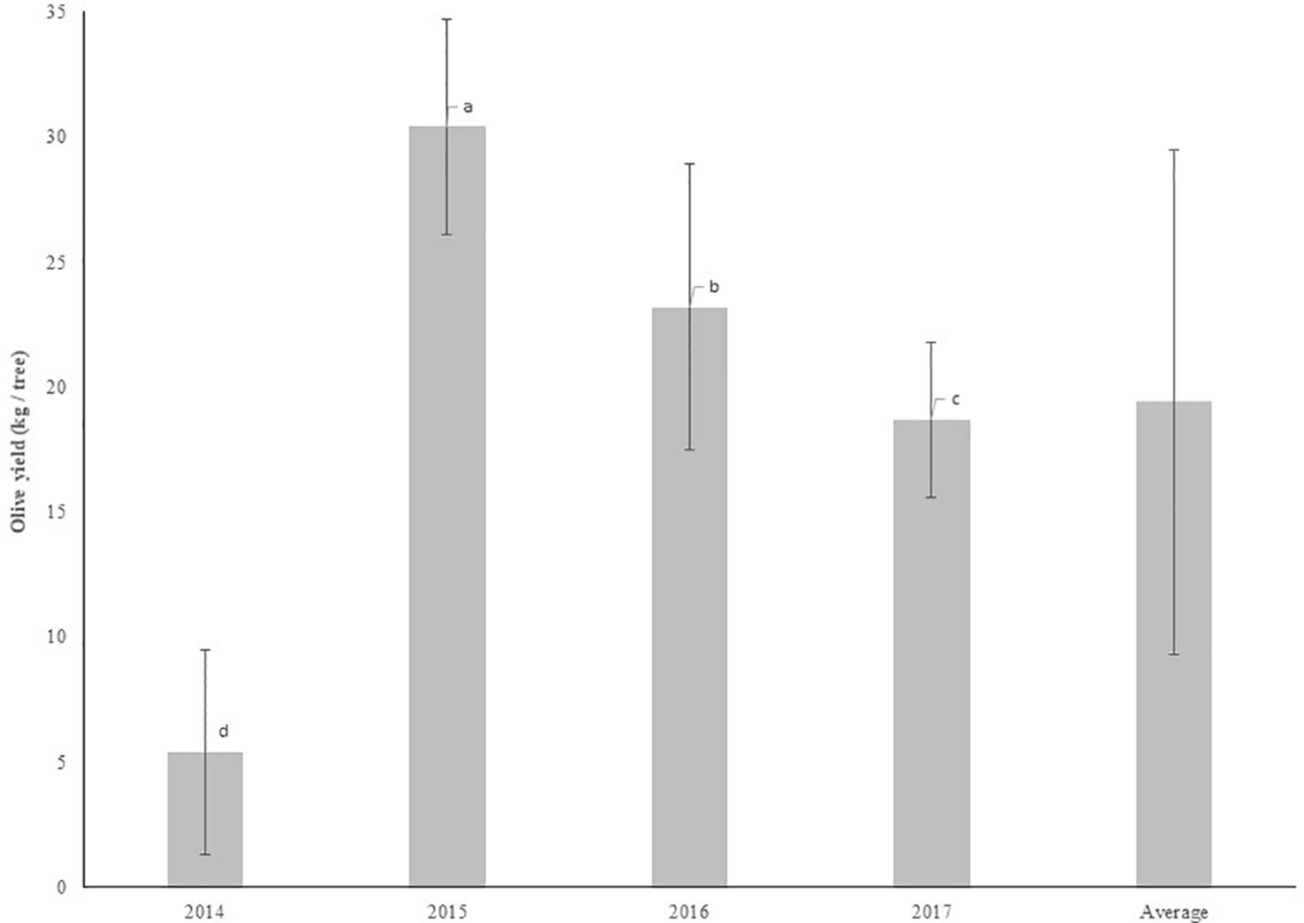
Olive yield by year (mean±sd). Legend: T1, treatment 1; T2, treatment 2; T3, treatment 3; T4, treatment 4. Columns followed by the same letter are not significantly different by Duncan multiple range test at the 5% level.

**Figure 11:**
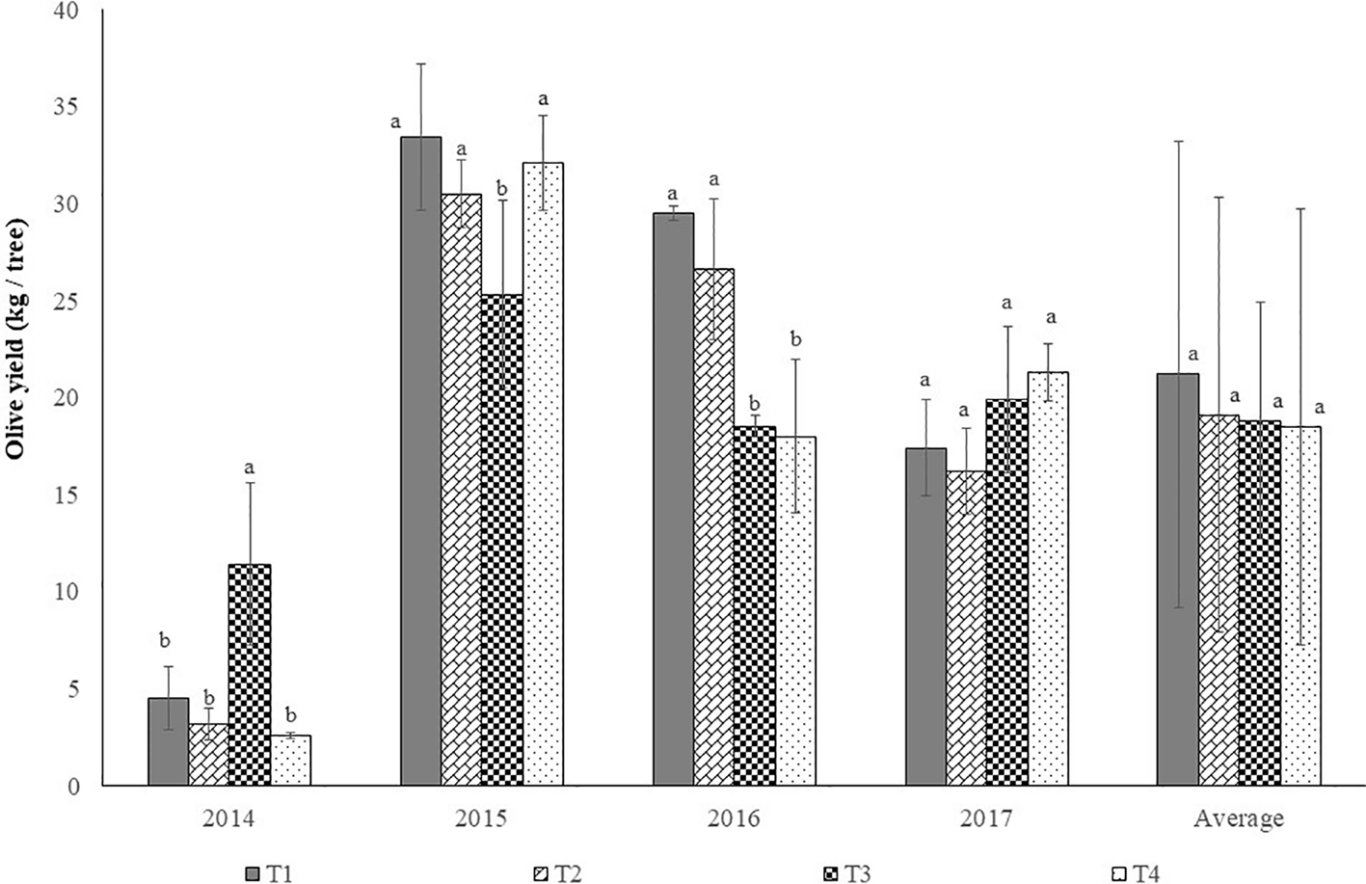
Olive yield by treatment in each year and the average yield of the trial (mean±sd). Legend: T1, treatment 1; T2, treatment 2; T3, treatment 3; T4, treatment 4. In each year and in average, columns followed by the same letter are not significantly different by Duncan multiple range test at the 5% level, with the exception of 2015, where p < 0.1.

**Figure 10** shows the average yield of olives per tree in each year. There were significant differences (P < 0.05) between the years, with the highest production achieved in 2015, which was significantly higher (P ≤ 0.05) than in the other years, which differed from each other. These results confirm the alternate bearing typical of this species, which can be changed with pruning interventions. After the decrease in production verified from 2015 to 2016, it would be expected that in 2017 there would be an increase in production. However, pruning interventions executed in 2017, with a reduction in the canopy volume and the elimination of potentially productive branches, resulted in a decrease in production compared to 2016. **Figure 11** shows olive yield by treatment in each year and the average yield of the trial. In 2014, significant differences (P < 0.05) were registered in olive yield per tree between treatments. Treatment 3 has revealed significantly (P ≤ 0.05) higher yield than the other treatments, as consequence of the smaller pruning intensity applied. The disk-saw pruning machine performs non-selective trimming of the canopy. In treatment T3, the significantly higher production may have been originated in productive branches issued in the previous year on the northwestern side of the canopy which was left uncut 2014. In treatments T2 and T4, the lateral cuts on both sides eliminated a considerable part of the productive branches issued in the previous year, diminishing productive potential in comparison with treatment T3. In the case of treatment T1, despite having been subjected to manual pruning, which has greater selectivity, the elimination of a considerable part of the canopy reduced the productive potential.

In 2015 significant differences in olive yield were registered between treatments (P < 0.05). Treatment 3 obtained a significantly lower yield (P ≤ 0.05) than the other treatments as a consequence of the higher yield obtained in the previous year. Given that the olive tree is characterized by an alternate bearing, a year with low production allows more vegetative growth. The greater leaf mass developed this year will boost higher yield the following year. This characteristic explains the considerable increase in production that occurred in treatments T1, T2, and T3 from 2014 to 2015. In the case of treatment T3 this increase was not so pronounced, since in 2014 the vegetative growth was conditioned by the existing tree production.

In 2016 significant differences (P < 0.05) were found in olive yield between treatments. Treatment 3 and treatment 4 show significantly (P ≤ 0.05) lower yield than treatments 1 and 2. The fall in production of treatment 3 compared to treatments 1 and 2 is associated with a reduction in canopy volume due to topping in summer 2015 and cutting in the northwest side in winter 2016, which left these trees with lower leaf mass and consequently with lower fruiting potential. High production in a smaller tree canopy (T4 in 2015) will tend to penalize the release of productive branches and consequently the production of 2016. In 2017 production shows the opposite trend to 2016, although without significant differences between treatments (P > 0.1). On average, no significant differences between treatments were found (P > 0.1)

These results show the potential of mechanical pruning as a method for reducing labor dependence, without significant negative influence in olive production, in line with the results obtained in traditional olive orchards by Pastor and Humanes (1998) Dias (2006); Dias et al. (2012), and Dias et al. (2014). The results obtained in this trial differ from those obtained by Ferguson et al. (2002) in an olive grove for table olives, who found that topping and hedging every 2 years causes a decrease in olive yield, particularly in the pruning year. This decrease in production did not affect the gross net return because the fruits being of a larger caliber benefited of a better market price.

The present work also seems to reveal that frequent mechanical pruning interventions tend to penalize olive yield. It appears desirable to perform a more intense mechanical pruning spaced in time, as recommended by Pastor and Humanes (1998) for high density olive groves.

Manual pruning complement to mechanical pruning did not increase olive yield in comparison to the obtained in trees with the absence of manual pruning complement.

Manual pruning complement to the mechanical pruning (T2), particularly in following years to the mechanical pruning, should be regarded as a potentially important technique, since it may contributed to higher yields as verified by Dias et al. (2014) on a traditional olive orchard after more than 10 years submitted to mechanical pruning.

### Harvester Efficiency

**Figure 12:**
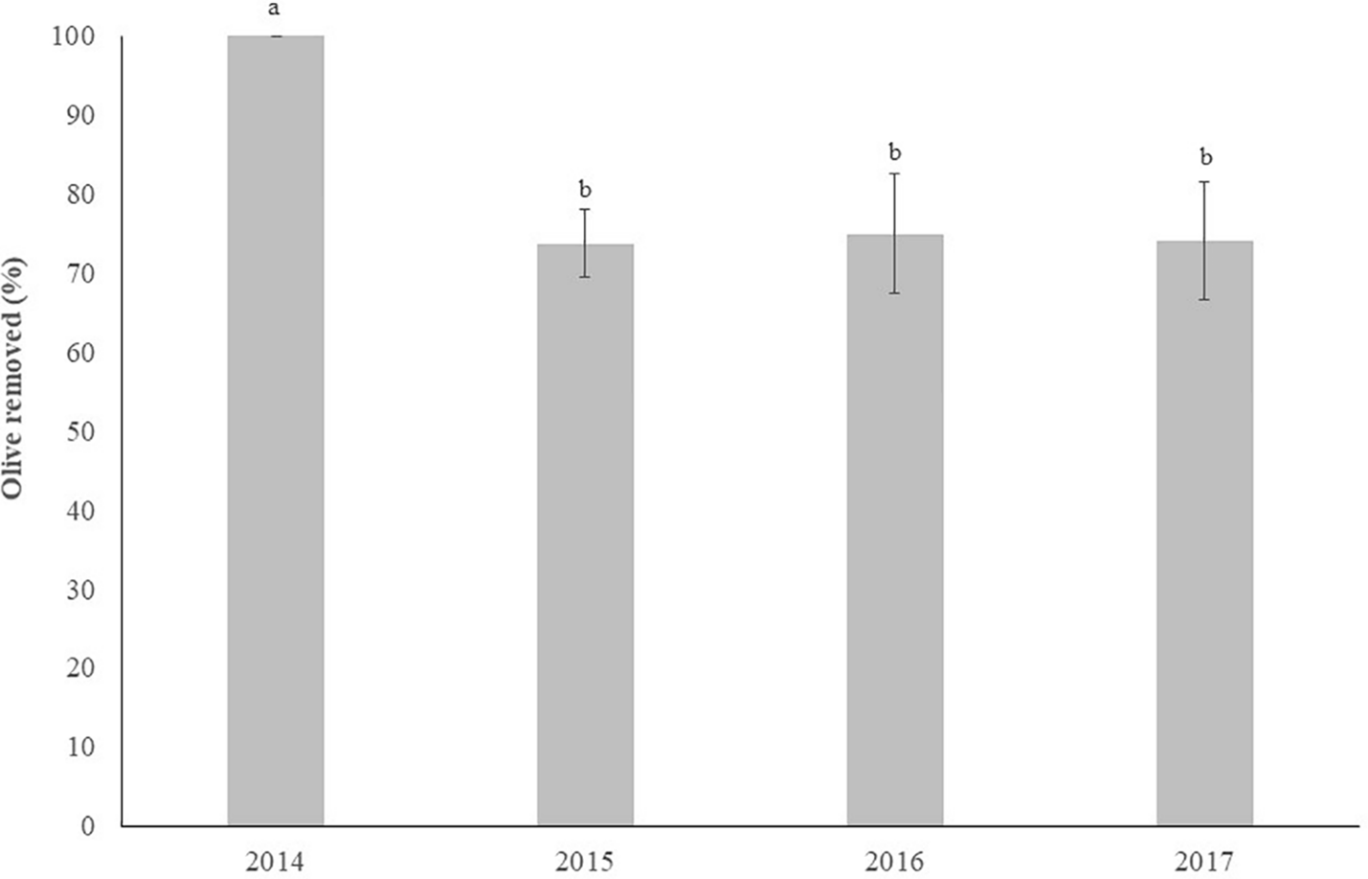
Effect of year in the harvester efficiency (mean±sd). Columns followed by the same letter are not significantly different by Duncan multiple range test at the 5% level.

**Figure 12** shows the removal efficiency of olives by the SRCCSH in each year. Significant differences (P < 0.05) were observed between the years, with the highest efficiency being achieved in 2014, which was significantly higher (P < 0.05) than those observed in the other years which didn’t differ from each other. It should be noted that the result obtained in 2014 had several constraints:

- low level of production;
- olives with anthracnose disease;
- olives at an advanced maturity stage (practically all black).

**Figure 13:**
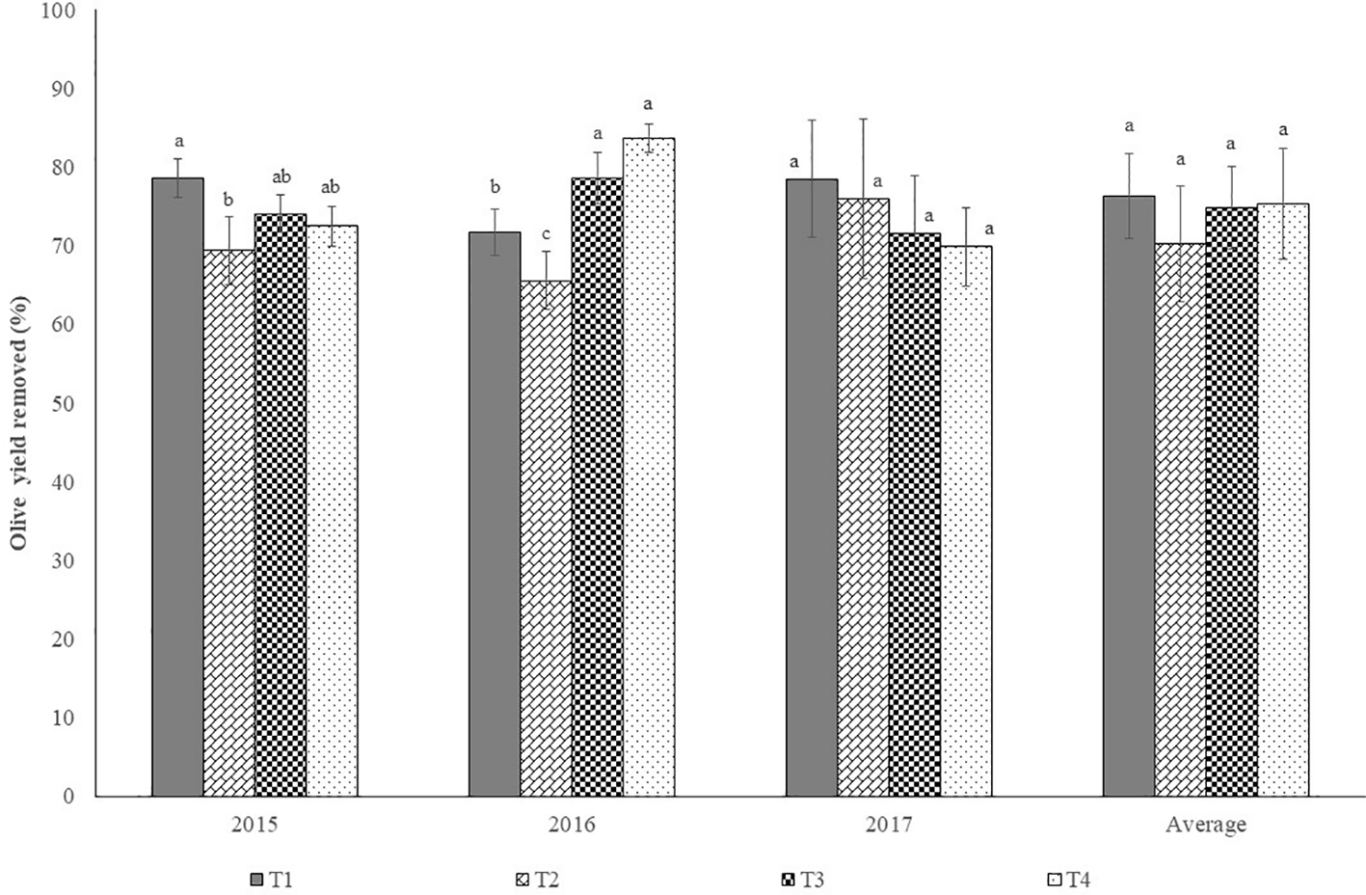
Harvester efficiency (%) by treatment, in each year and the average of the trial (mean±sd). Legend: T1, treatment 1; T2, treatment 2; T3, treatment 3; T4, treatment 4. Columns followed by the same letter are not significantly different by Duncan multiple range test at the 5% level.

**Figure 13** shows harvester efficiency per treatment in each year and the average efficiency over the period 2015 to 2017.

In 2017, **Figures 7** and **9** show that, before pruning, treatments T1, T2, and T4 had canopies of similar size, leading to admit the same at 2016 harvest. However, harvest efficiency was significantly higher in T4 than in the other treatments. Possibly the significantly lower yield of T4 relative to T1 and T2, may be a justification, since the same detaching vibrating energy is available for fewer fruits. This could also justify the significantly higher value of efficiency of treatment T3 relative to treatments T1 and T2. Ferguson and Castro-García, (2014) also obtained significantly higher harvesting efficiency on trees with lower yields compared to those submitted to manual pruning that were harvested by hand. However, these results refer to only 1 year and were obtained from trees intended for the production of table olives. In 2017 no significant differences (P > 0.1) were found between treatments in harvest efficiency. Although no data is available concerning the regrowth after 2017 pruning, **Figures 7** and **9** reveal that canopy size was left with similar volume in all treatments. Taking in account that no significant differences were found in olive yield among treatments (**Figure 11**), this may justify that no significantly differences were also found in harvest efficiency.

In 2015 significant differences (P < 0.05) were found between treatments in harvest efficiency. At the 2015 harvest, and assuming that the size of the trees is revealed by their dimensions in 2016 (**Figure 7**), it can be appreciated that trees of T3 and T4 are lower than the trees of T1 and T2.

At harvest olives were below the 4 m height, whereas in treatments T1 and T2 production was above the 4 m mark. However, the results of harvest efficiency in 2015 reveal that more factors not counted for at the present work have to be considered. Fruit detachment force and fruit weight presented a low correlation with harvest efficiency (Gil-Ribes et al., 2011). The spatial distribution of the olive in the canopy, associated with the trajectory described by the active organs of the vibrating mast, may assume an increasing importance when deciding pruning interventions to enhance harvest efficiency without jeopardizing yields and at contained costs. The influence of the manual intervention done in 2014 in T1 and T2, may be still influence the transmission of vibrating energy to the canopy in the following years, especially in 2015. Also, it is not understood why, similar canopies as in T3 and T4, bearing significantly different yield capacity, showed also similar harvest efficiency.

On average (period 2015 to 2017), no significant differences (P > 0.05) among treatments in harvest efficiency were found.

## Conclusions

In the period of 4 years, on average, there were no significant differences (P ≤ 0.05) between treatments in olive production.

In view of this fact:

- the cost with only mechanical pruning is lower than pruning with manual interventions.
- manual complement pruning was not relevant since it did not lead to increased production and led to an increase in pruning costs, as had already occurred in the traditional olive grove trial. Complementary manual pruning interventions only make sense to eliminate excess wood accumulated over a relatively long period of time and should be carried out sporadically. Two supplementary annual pruning interventions over a 4-year period are not advisable.
- the option for pruning with a disk machine on the side faces in alternate years has also not been interesting since it does not reduce the pruning costs, it does not enhance production of olives. Carrying out the cuts on the sides of the canopy more frequently, while allowing the canopy volume to be controlled, does not allow for the full production potential of the regrowth that appear after the cuts on the side faces.

In terms of the influence of pruning on the performance of the SRCCSH, there were no significant differences between treatments.

## Data Availability Statement

All datasets generated for this study are included in the article/supplementary material.

## Author Contributions

AD conceived and designed the experiment. AD, JF, AP, and JP performed the evaluation. AD and JP analyzed the data and wrote the paper.

## Funding

This work is funded by National Funds through FCT—Foundation for Science and Technology under the Project UID/AGR/00115/2013 and by Portuguese Agriculture Ministry research program PRODER under the project 55344.

## Conflict of Interest

The authors declare that the research was conducted in the absence of any commercial or financial relationships that could be construed as a potential conflict of interest.
